# Apigenin, a Partial Antagonist of the Estrogen Receptor (ER), Inhibits ER-Positive Breast Cancer Cell Proliferation through Akt/FOXM1 Signaling

**DOI:** 10.3390/ijms22010470

**Published:** 2021-01-05

**Authors:** Thu Ha Pham, Yann Le Page, Frédéric Percevault, François Ferrière, Gilles Flouriot, Farzad Pakdel

**Affiliations:** Inserm, EHESP, Irset (Institut de Recherche en Santé, Environnement et Travail)-UMR_S1085, Rennes University, F-35000 Rennes, France; thu-ha.pham@univ-rennes1.fr (T.H.P.); yann.le-page@univ-rennes1.fr (Y.L.P.); frederic.percevault@univ-rennes1.fr (F.P.); francois.ferriere@univ-rennes1.fr (F.F.); gilles.flouriot@univ-rennes1.fr (G.F.)

**Keywords:** breast cancer, endocrine resistance, ER, Akt, FOXM1, phytochemicals, apigenin

## Abstract

Approximately 80% of breast cancer (BC) cases express the estrogen receptor (ER), and 30–40% of these cases acquire resistance to endocrine therapies over time. Hyperactivation of Akt is one of the mechanisms by which endocrine resistance is acquired. Apigenin (Api), a flavone found in several plant foods, has shown beneficial effects in cancer and chronic diseases. Here, we studied the therapeutic potential of Api in the treatment of ER-positive, endocrine therapy-resistant BC. To achieve this objective, we stably overexpressed the constitutively active form of the Akt protein in MCF-7 cells (named the MCF-7/Akt clone). The proliferation of MCF-7/Akt cells is partially independent of estradiol (E2) and exhibits an incomplete response to the anti-estrogen agent 4-hydroxytamoxifen, demonstrating the resistance of these cells to hormone therapy. Api exerts an antiproliferative effect on the MCF-7/Akt clone. Api inhibits the proliferative effect of E2 by inducing G2/M phase cell cycle arrest and apoptosis. Importantly, Api inhibits the Akt/FOXM1 signaling pathway by decreasing the expression of FOXM1, a key transcription factor involved in the cell cycle. Api also alters the expression of genes regulated by FOXM1, including cell cycle-related genes, particularly in the MCF-7/Akt clone. Together, our results strengthen the therapeutic potential of Api for the treatment of endocrine-resistant BC.

## 1. Introduction

Breast cancer (BC) is the most commonly diagnosed cancer and the leading cause of cancer-related death in women worldwide; nearly 2.1 million new cases were diagnosed in 2018 [1]. Among the different types of BC, estrogen receptor (ER)-positive subtypes, namely, Luminal A and Luminal B, represent approximately 80% of cases [2]. These subtypes tend to be less immediately aggressive than other subtypes and can initially respond to endocrine treatment [2]. However, 15 to 20% of these tumors are intrinsically resistant to treatment, and 30 to 40% acquire resistance to treatment over a period of several years [3]. Approximately 40% of patients with BC acquire endocrine resistance following therapy with tamoxifen [4]. One of the important mechanisms by which endocrine resistance is acquired is alterations in the PI3K/Akt/mTOR pathway [5,6]. The activation of Akt, the key protein in this pathway, is correlated with hormone resistance and predicts a worse outcome in endocrine therapy-treated patients [7,8]. The Akt1-E17K mutation has been observed in 4 to 8% of BC patients, can lead to Akt activation and contribute to mammary tumorigenesis [9]. Therefore, Akt is an important therapeutic target, in order to identify new treatments for endocrine-resistant BC.

The PI3K/Akt signaling pathway is one of the upstream kinase pathways that regulate Forkhead box protein M1 (FOXM1), which is an essential proliferation-associated transcription factor that regulates the cell cycle [10,11]. FOXM1 regulates all the hallmarks of cancer, including proliferation, mitosis, EMT, invasion, and metastasis [10]. In ER-positive BC, increased expression of FOXM1 is associated with increased cell invasiveness and resistance to endocrine treatments. Consequently, increased FOXM1 expression is correlated with significantly reduced survival in BC patients [12]. Akt can promote the activity of FOXM1 by blocking the activity of FOXO3a, which antagonizes the activity of FOXM1 [13]. Therefore, the FOXO3a-FOXM1 axis is actually a crucial therapeutic target for the treatment of cancer, particularly for overcoming drug resistance [14].

BC is a multifactorial disease, and certain known factors, including diet, can increase or decrease the risk of contracting the disease [15,16,17]. Epidemiological studies and systematic analyses suggest that diets rich in flavonoids are inversely associated with a risk of BC [18,19,20,21]. Among these flavonoids, apigenin (4′,5,7-trihydroxyflavone) (Api) is one of the most frequently studied phenolic molecules because it is very widely expressed in the plant kingdom and it is common in teas, dry herbs, fruits, and vegetables [22]. Api is abundantly present in celery, parsley, and dried chamomile flowers [22] as well as in herbs that have been used in traditional Chinese medicine for centuries [23]. Recent studies have shown that Api can inhibit the growth of BC cells and xenograft tumors both, in hormone receptor-positive BC [24,25,26,27] and in triple-negative breast cancer (TNBC) [28,29,30,31]. In hormone receptor-positive BC, Api can block the expression of mucin 1 C-terminal subunit oncoprotein [24] and induce apoptosis by activating p53 and inhibiting STAT3 and NFκB [26]. In TNBC, Api suppresses stem cell-like properties by inhibiting YAP/TAZ activity [30] and blocks IL-6 expression [31]. Moreover, Api can inhibit the mechanisms of drug resistance and enhance the efficiency of chemotherapy [32,33,34,35]. Therefore, Api may be a potential treatment or adjuvant for endocrine-resistant BC.

In the current study, we investigated the effect of Api in ER-positive MCF-7 (MCF-7/Ctrl) BC cells and Akt-activated MCF-7 (MCF-7/Akt) BC cells. We observed that in addition to the antagonistic effect of Api on E2-induced proliferation, treatment with Api alone decreased MCF-7/Akt cell proliferation. This effect of Api enhanced the effect of 4-hydroxytamoxifen (4-OHT) when the two agents were used in combination. Regarding ER signaling, Api exerted an agonist/antagonist effect on ER transcriptional activity and on ER target gene expression. Regarding Akt/FOXO3a/FOXM1 signaling, the expression of FOXO3a was suppressed and the expression of FOXM1 was enhanced in MCF-7/Akt cells compared with MCF-7/Ctrl cells. Api treatment decreased the protein expression of FOXM1 and its related genes, especially in the MCF-7/Akt clone. Together, our results support the therapeutic potential of Api in BC, particularly in endocrine-resistant BC.

## 2. Results

### 2.1. Construction and Characterization of Akt-Activated MCF-7 (MCF-7/Akt) Cells and MCF-7/Ctrl Cells

**Figure 1:**
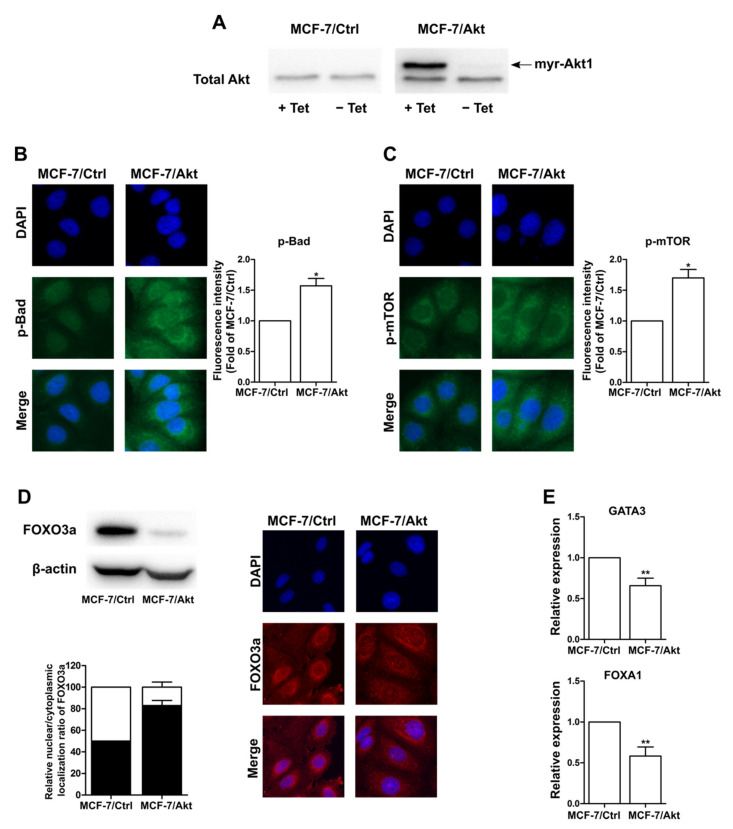
Characterization of MCF-7 cell clones expressing wild-type Akt (MCF-7/Ctrl) and its constitutively active form (MCF-7/Akt). (**A**) Equal amounts of protein extracts from MCF-7/Ctrl cells and MCF-7/Akt cells treated with or without tetracycline (Tet) were analyzed by Western blotting with an antibody specific for Akt. Notably, myr-Akt1 has a larger molecular mass than wild-type Akt1. (**B**,**C**) Protein expression and subcellular localization of phospho-Bad (**B**) and phospho-mTOR (**C**) in MCF-7/Ctrl cells and MCF-7/Akt cells, as examined by immunofluorescence. (**D**) Equal amounts of protein extracts from MCF-7/Ctrl cells and MCF-7/Akt cells were analyzed by Western blotting with an antibody specific for FOXO3a (left, above). Protein expression and subcellular localization of FOXO3a in MCF-7/Ctrl cells and MCF-7/Akt cells examined by immunofluorescence (right). Quantification of the nuclear/cytoplasmic localization ratio of FOXO3a (left, below). Fluorescence intensity in the nucleus and cytoplasm of each cell was measured using ImageJ software. The median nuclear/cytoplasmic localization ratio of the control condition was used as a threshold to determine the percentage of cells below (black) and above (white) this threshold in each condition. (**E**) Gene expression of GATA3 (above) FOXA1 and (below) in MCF-7/Ctrl cells and MCF-7/Akt cells, as revealed by RT-PCR. Statistical analyses were performed with a Mann-Whitney test. * *p*-value < 0.05, ** *p*-value < 0.01 is considered to be significantly different from the MCF-7/Ctrl cell group.

As described in the Methods section, we constructed MCF-7 cells in which Akt was constitutively active (MCF-7/Akt cells) by transfecting parent MCF-7 cells with the myr-Akt1 cDNA vector using the T-RexTM system. The myr-Akt1 construct contains the 11 N-terminal amino acids of the avian sequence fused in frame to the N-terminus of the wild-type Akt coding sequence. These additional amino acids, which are required for protein myristoylation, allow the attachment of Akt to the intracellular side of the cell membrane. Therefore, Akt can be activated without the activation of its upstream proteins. Western blot analyses using an anti-Akt antibody revealed that the MCF-7/Akt cells treated with tetracycline strongly expressed constitutively active Akt, which has a larger molecular mass than wild-type Akt due to the 11 additional amino acids (Figure 1A).

Next, we examined the expression and subcellular localization of the two proteins downstream of Akt, namely, phospho-Bad and phospho-mTOR, using immunofluorescence. We observed an increase in the phospho-Bad and phospho-mTOR levels in the MCF-7/Akt cells compared with the MCF-7/Ctrl cells (Figure 1B,C). Moreover, the expression of these proteins was almost increased in the cytoplasm. On the other hand, it is well documented that the Akt-mediated phosphorylation of FOXO proteins induces their cytoplasmic translocation and degradation [13]. Thus, we evaluated the protein level and nuclear/cytoplasmic localization of FOXO3a in the two MCF-7 clones. As shown by the Western blot analyses in Figure 1D, we observed a sharp decline in the protein expression of FOXO3a in the MCF-7/Akt cells. Moreover, immunofluorescence analyses showed a decrease in the ratio of nuclear/cytoplasmic localization of FOXO3a in the MCF-7/Akt cells compared to the MCF-7/Ctrl cells (Figure 1D). Furthermore, we observed a decrease in the gene expression of two pioneer transcription factors, GATA3 and FOXA1, in the MCF-7/Akt cells (Figure 1E). Since these factors can associate with compacted chromatin to facilitate the binding of additional transcription factors, including ER [36], the decrease in these factors in the MCF-7/Akt cells may alter ER activity and enhance cell transformation.

Overall, we confirmed the constitutive activation of Akt in MCF-7/Akt cells. The results also show that activation of Akt negatively regulates FOXO3a by significantly decreasing its protein levels and increasing its exclusion from the nucleus and its presence in the cytoplasm.

### 2.2. Effects of Api on the Proliferation of MCF-7/Ctrl Cells and MCF-7/Akt Cells

**Figure 2:**
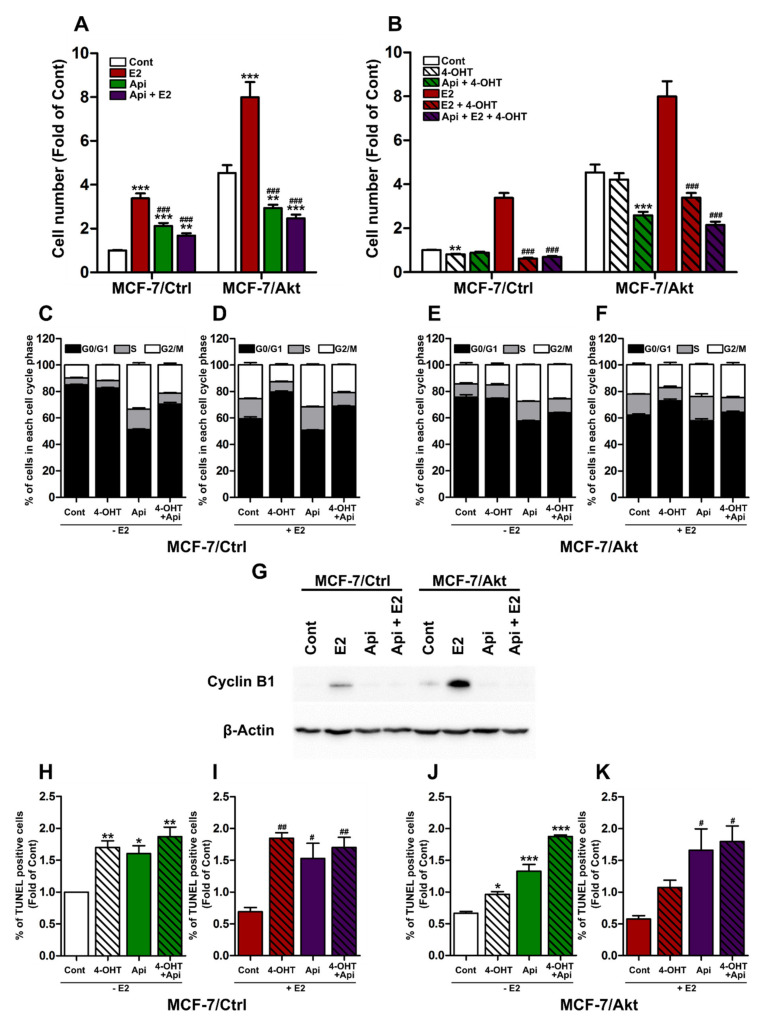
Effects of apigenin on the proliferation and cell cycle of MCF-7/Ctrl cells and MCF-7/Akt cells. (**A**) MCF-7/Ctrl cells and MCF-7/Akt cells were treated with solvent (0.1% (*v*/*v*) DMSO and 0.1% (*v*/*v*) ethanol) as the control (Cont), 1 nM estradiol (E2), or 10 µM apigenin (Api) alone or in combination with E2. (**B**) MCF-7/Ctrl cells and MCF-7/Akt cells were treated with solvent (Cont) or 1 µM 4-hydroxytamoxifen (4-OHT) alone or in combination with Api in the presence or absence of E2. Cell numbers were determined using the sulforhodamine B assay after 6 days of treatment. The experiment was conducted 3 times in quintuplicate. The results are expressed as the fold change in cell number compared with that in the control MCF-7/Ctrl cells and are presented as the mean ± SEM. For the cell cycle assays, MCF-7/Ctrl cells (**C**,**D**) and MCF-7/Akt cells (**E**,**F**) were treated with solvent (Cont), 4-OHT and Api alone or in combination in the presence or absence of E2 for approximately 24 h. The cell cycle was analyzed by flow cytometry after propidium iodide staining. The experiment was conducted 3 times. The results are expressed as the percentage of cells in each phase of the cell cycle and are presented as the mean ± SEM. (**G**) Equal amounts of protein extracts from MCF-7/Ctrl cells and MCF-7/Akt cells were analyzed by Western blotting with antibodies specific for cyclin B1 and β-actin. MCF-7/Ctrl cells and MCF-7/Akt cells were treated with solvent (Cont), E2 and Api alone or in combination with E2 for 24 h. For apoptosis analysis, MCF-7/Ctrl cells (**H**,**I**) and MCF-7/Akt cells (**J**,**K**) were treated with solvent (Cont), 4-OHT and Api alone, or in combination, in the presence or absence of E2 for approximately 24 h. Apoptotic cells were detected using terminal deoxynucleotidyl dUTP nick end labeling (TUNEL) assays. The experiment was conducted 3 times. The percentage of apoptotic cells was quantified and expressed as the fold change of the Cont-treated of the MCF-7/Ctrl clone. The results are presented as the mean ± SEM. Statistical analyses were performed with one-way ANOVA followed by Tukey’s post hoc test. * *p*-value < 0.05, ** *p*-value < 0.01, *** *p*-value < 0.001 indicate significant differences from the control group in the same clone. # *p*-value < 0.05, ## *p*-value < 0.01, ### *p*-value < 0.001 indicate significant differences from the E2 group in the same clone.

To determine the effect of Api on cell proliferation, MCF-7/Ctrl cells and MCF-7/Akt cells were cultured in the presence or absence of Api alone or Api with E2 (Figure 2A). Compared with the MCF-7/Ctrl cells, which proliferate very slowly in the absence of E2, the MCF-7/Akt cells grew independently of E2. Then, the proliferation of the MCF-7/Ctrl cells increased by nearly four-fold with E2 treatment, while the proliferation of the MCF-7/Akt cells increased by less than two-fold. Compared with solvent alone, Api alone increased the proliferation of the MCF-7/Ctrl cells, but interestingly, it decreased the proliferation of the MCF-7/Akt cells. On the other hand, the combination of Api and E2 decreased the E2-induced proliferation of both the MCF-7/Ctrl cells and MCF-7/Akt cells.

Next, we compared the effect of 4-hydroxytamoxifen (4-OHT) alone or 4-OHT in combination with Api, in the presence or absence of E2, in the two clones (Figure 2B). We observed that in the MCF-7/Ctrl cells, 4-OHT alone slightly but significantly inhibited proliferation compared with solvent alone. Moreover, 4-OHT antagonized the effect of E2 on cell proliferation. The effect of 4-OHT was not altered when the MCF-7/Ctrl cells were also treated with Api. However, the baseline proliferation of the MCF-7/Akt cells was not affected by 4-OHT treatment. However, the combination of 4-OHT and Api enhanced the antiproliferative effect of 4-OHT in this clone. Together, our results confirm that the constitutive activation of Akt in MCF-7 cells induces the emergence of hormone resistance that is characterized by autonomous growth and the acquisition of characteristics that render MCF-7 cells resistant to 4-OHT. Api partially reduces this resistance by enhancing the antiproliferative effect of 4-OHT.

Since the cell cycle plays an important role in cell proliferation, we assessed the effects of all the treatments on the cell cycle in both MCF-7 clones. The percentage of cells in each phase of the cell cycle was determined by flow cytometry after propidium iodide staining. In the MCF-7/Ctrl cells, approximately 85% of the cells were observed to be in the G0/G1 phase, 5% in the S phase, and 10% in the G2/M phase (Figure 2C). However, in the MCF-7/Akt cells, the percentage of cells in the S phase was two times higher, and 15% of the cells were observed to be in the G2/M phase (Figure 2E). As expected, E2 treatment increased the proportion of cells in the S and G2/M phases in the two MCF-7 clones, and the fold change was greater in the MCF-7/Ctrl cells than in the MCF-7/Akt cells (Figure 2D,F). Surprisingly, Api treatment also exerted agonistic effects similar to those observed with E2 treatment by increasing the percentage of cells in the S and G2/M phases in both clones, although overall, the increase in the cells in the G2/M phases induced by Api was more prominent than that induced by E2 (Figure 2C–F). The 4-OHT treatment decreased the percentage of cells in the S and G2/M phases and antagonized the effects of E2 and Api on the cell cycle when used in combination with these treatments (Figure 2C–F).

To further distinguish the effect of Api from that of E2 on the cells in the G2/M phases, we examined the effects of these treatments on the expression of a marker of these cell phases, namely, cyclin B1, which is an essential protein for the G2/M phases (Figure 2G). While E2 clearly increased the expression of this protein in the two clones, Api treatment alone did not change the expression of cyclin B1 in the MCF-7/Ctrl cells and decreased its expression in the MCF-7/Akt cells. When the cells were treated with Api in combination with E2, Api blocked the effects of E2. Since cyclin B1 plays an essential role in the G2/M phase transition by enhancing mitochondrial function in order to provide energy for this transition [37], deficiency of cyclin B1 under Api treatment may cause a reduction in the number of cells in the M phase. These results could partially explain why both E2 and Api increase the number of cells in the S and G2/M phases, but E2 exerts proliferative effects and Api exerts anti-proliferative effects.

Since the cell number is the result of cell growth and cell death, we next analyzed the effects of Api on apoptosis (Figure 2H–K). We observed that the baseline percentage of apoptotic cells in the MCF-7/Ctrl (Figure 2H) was higher than that in the MCF-7/Akt cells (Figure 2J). As expected, E2 treatment decreased the percentage of apoptotic cells in the MCF-7/Ctrl, while the effect of E2 was much weaker in the MCF-7/Akt cells. More importantly, treatment with 4-OHT or Api increased the percentage of apoptotic cells in the two cell models in the presence or absence of E2 (Figure 2H–K). The combination of 4-OHT and Api in absence of E2 had a synergistic effect on the MCF-7/Akt cells (Figure 2J), which was consistent with the results of the cell counts (Figure 2B).

Together, our results confirmed the anti-proliferative effects of Api in E2-dependent MCF-7/Ctrl cells and in partially E2-independent MCF-7/Akt cells. These effects are due to the Api-induced G2/M phase arrest and apoptosis.

### 2.3. Effects of Apigenin on ER Genomic Activity

**Figure 3:**
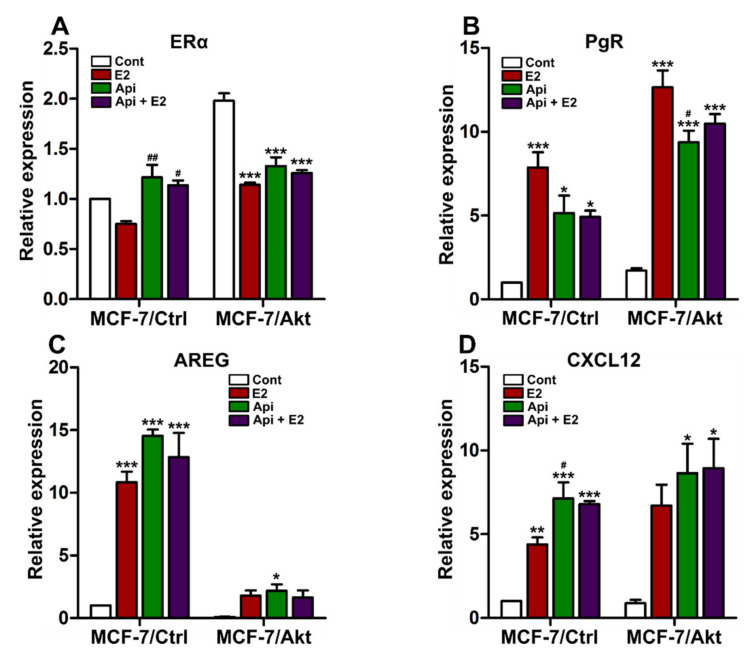
Effect of apigenin on ER target genes. MCF-7/Ctrl cells and MCF-7/Akt cells were treated with 0.1% (*v*/*v*) DMSO and 0.1% (*v*/*v*) ethanol as the control (Cont), 1 nM estradiol (E2), or 10 µM apigenin (Api) alone or in combination with E2 for 24 h. The gene expression of estrogen receptor 1 (ERα) (**A**), progesterone receptor (PgR) (**B**), amphiregulin (AREG) (**C**) and chemokine (C-X-C motif) ligand 12 (CXCL12) (**D**) was assessed by real-time PCR and normalized to the expression of the housekeeping genes GAPDH and TBP. The experiment was conducted three times in triplicate. The results are expressed as the fold change in gene expression compared with that in the control of the MCF-7/Ctrl cells and are presented as the mean ± SEM. Statistical analyses were performed with one-way ANOVA followed by Tukey’s post hoc test. * *p*-value < 0.05, ** *p*-value < 0.01, *** *p*-value < 0.001 indicate significant differences from the control group in the same clone. # *p*-value < 0.05, ## *p*-value < 0.01 indicate significant differences from the E2 group in the same clone.

To better understand the molecular mechanisms involved in the anti-proliferative effect of Api on the two clones, we first investigated the effects of Api on ER signaling. At the gene expression level, ERα expression was increased in the MCF-7/Akt clone compared to the MCF-7/Ctrl clone. E2 treatment decreased ERα gene expression in the two clones, and cotreatment with Api completely attenuated the effect of E2 in the MCF-7/Ctrl cells, but the effect was not significant in the MCF-7/Akt cells (Figure 3A). In relation to the effects of Api on ER target gene expression, Api exerted an agonist/antagonist effect on the gene expression of PgR (Figure 3B). However, Api exerted a complete agonist effect on the gene expression of AREG (Figure 3C) and CXCL12 (Figure 3D). Comparing the two clones, the baseline level of PgR increased in the MCF-7/Akt cells compared to the MCF-7/Ctrl cells. Notably, the baseline level of AREG dramatically decreased in the MCF-7/Akt cells.

**Figure 4:**
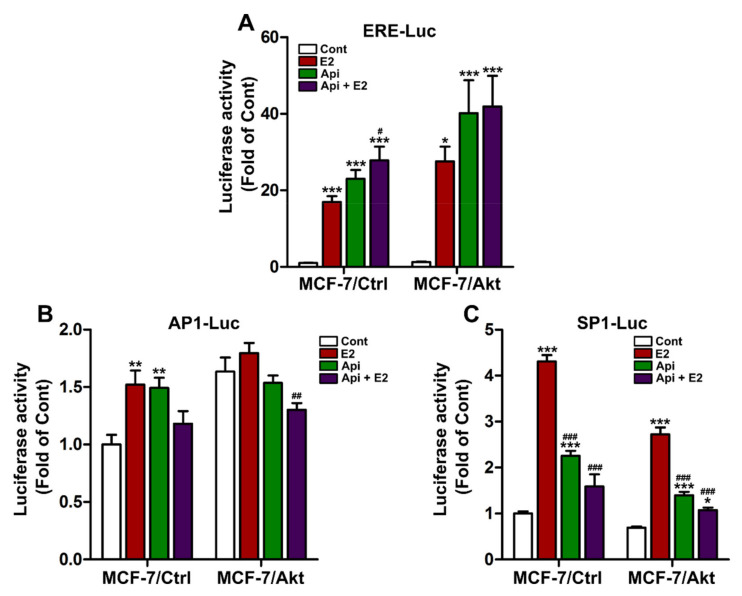
Effects of apigenin on the ER genomic activity. MCF-7/Ctrl cells and MCF-7/Akt cells were transiently transfected with an estrogen-responsive element luciferase reporter plasmid (ERE-Luc) (**A**), activator protein 1 luciferase reporter plasmid (AP1-Luc) (**B**) or specificity protein 1 luciferase reporter plasmid (SP1-Luc) (**C**) as well as a CMV-β-galactosidase plasmid as a control to assess transfection efficiency. Then, the cells were treated with 0.1% (*v*/*v*) DMSO and 0.1% (*v*/*v*) ethanol as the control (Cont), 1 nM estradiol (E2), or 10 µM apigenin (Api) alone or in combination with E2 for 24 h. The experiment was conducted 3 times in triplicate. The results are expressed as the fold change in luciferase activity compared with that in the control of the MCF-7/Ctrl cells and are presented as the mean ± SEM. Statistical analyses were performed with one-way ANOVA followed by Tukey’s post hoc test. * *p-*value < 0.05, ** *p*-value < 0.01, *** *p*-value < 0.001 indicate significant differences from the control group in the same clone. # *p*-value < 0.05, ## *p*-value < 0.01, ### *p*-value < 0.001 indicate significant differences from the E2 group in the same clone.

To further understand why Api has agonist/antagonist effects on the expression of ER-dependent genes but complete agonist effects on other ER-dependent genes, we investigated the effects of Api on ER activation via different reporter genes using luciferase assays. Api exerted complete agonist effects on the estrogen-responsive element (ERE)-luciferase reporter gene in the absence and presence of E2 (Figure 4A). However, Api exerted an agonistic effect when used alone, but an antagonistic effect when used in combination with E2 on the activation of activator protein 1 (AP1)- and specificity protein 1 (SP1)-luciferase reporter genes (Figure 4B,C). These results indicate that the antagonistic effect of Api on the ER pathway when combined with E2 could be related to the action of ER on the AP1 and SP1 pathways, but not the ERE. Of note, a similar pattern of luciferase activity was also observed in both clones.

### 2.4. Effects of Apigenin on the Expression of FOXM1 and FOXM1-Related Genes

**Figure 5:**
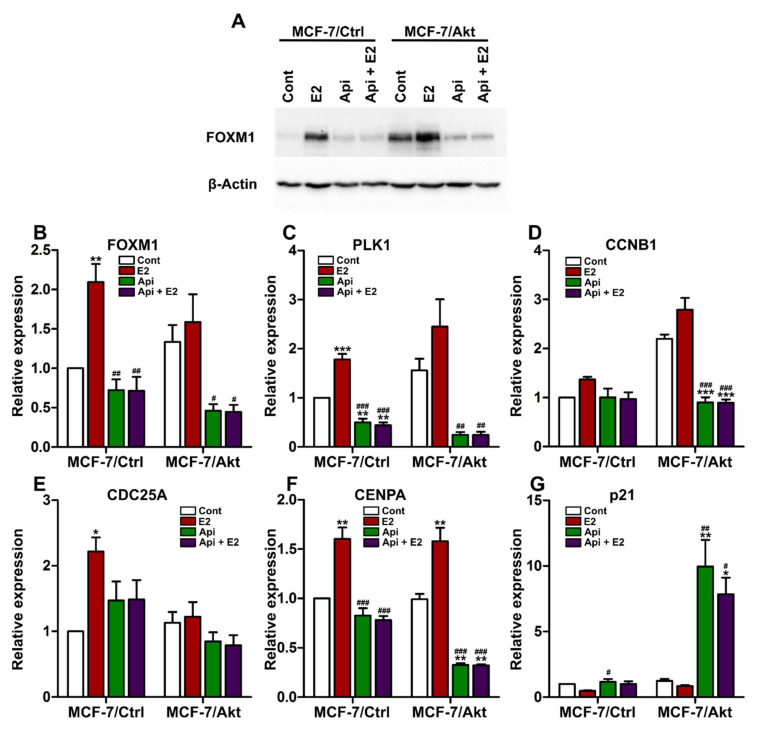
Apigenin inhibits the expression of FOXM1 and modulates the expression of FOXM1-related cell cycle genes. MCF-7/Ctrl cells and MCF-7/Akt cells were treated with 0.1% (*v*/*v*) DMSO and 0.1% (*v*/*v*) ethanol as the control (Cont), 1 nM estradiol (E2), or 10 µM apigenin (Api) alone or in combination with E2 for 24 h. (**A**) Equal amounts of protein extracts from MCF-7/Ctrl cells and MCF-7/Akt cells were analyzed by Western blotting with antibodies specific to FOXM1 and β-actin. The gene expression of forkhead box M1 (FOXM1) (**B**), polo-like kinase 1 (PLK1) (**C**), cyclin B1 (CCNB1) (**D**), cell division cycle 25A (CDC25A) (**E**), centromere protein A (CENPA) (**F**) and cyclin-dependent kinase inhibitor 1A (CDKN1A/p21) (**G**) was assessed by real-time PCR and normalized to the expression of the housekeeping genes GAPDH and TBP. The experiment was conducted three times in triplicate. The results are expressed as the fold change in gene expression compared with that in the control of the MCF-7/Ctrl cells and are presented as the mean ± SEM. Statistical analyses were performed with one-way ANOVA followed by Tukey’s post hoc test. * *p*-value < 0.05, ** *p*-value < 0.01, *** *p*-value < 0.001 indicate significant differences from the control group in the same clone. # *p*-value < 0.05, ## *p*-value < 0.01, ### *p*-value < 0.001 indicate significant differences from the E2 group in the same clone.

It has been well-demonstrated that FOXM1, a transcriptional regulator of G1/S progression, is also crucial for G2/M transition [38,39]. In addition, FOXO3a is a negative regulator of the expression and activity of FOXM1. In fact, FOXO3a behaves as a tumor suppressor, while FOXM1 is a potent oncogene that is widely overexpressed in endocrine-resistant cancers [13,14]. Since FOXO3a protein expression and nuclear localization were altered in the MCF-7/Akt cells, we examined the effect of Api on FOXM1. As demonstrated by Western blot analyses, the FOXM1 protein level was clearly increased in the MCF-7/Akt cells compared to the MCF-7/Ctrl cells (Figure 5A). E2 treatment strongly increased the FOXM1 protein expression level in both the MCF-7 cell clones, and interestingly, cotreatment with Api markedly reduced the effect of E2 (Figure 5A). Notably, compared to the control treatment, Api treatment alone decreased the FOXM1 protein level in the MCF-7/Akt cells, in which the baseline expression of FOXM1 was much higher than that in the MCF-7/Ctrl cells. Moreover, E2 treatment enhanced the translocation of FOXM1 from the cytoplasm to the nucleus, while cotreatment with Api reversed the effect of E2 (Supplementary Figure S2).

Similar to the protein expression, the baseline gene expression of FOXM1 was higher in the MCF-7/Akt cells than in the MCF-7/Ctrl cells (Figure 5B). Compared with the control, E2 treatment increased the expression of FOXM1 in the MCF-7/Ctrl cells, but this effect was not significant in the MCF-7/Akt cells. More importantly, Api treatment decreased the expression of FOXM1 when administered alone and antagonized the effect of E2 when administered in combination with E2. Notably, the inhibitory effect of Api on FOXM1 gene expression in the MCF-7/Akt cells was stronger than that in the MCF-7/Ctrl cells.

The cell cycle checkpoints, especially at the G1/S and G2/M phases, are mediated by FOXM1 via the regulation of key cell cycle-associated genes, such as polo-like kinase 1 (PLK1), cyclin B1 (CCNB1), cell division cycle 25A (CDC25A), centromere protein A (CENPA), and cyclin-dependent kinase inhibitor 1A (CDKN1A, also called p21). These genes are known to be transcriptional targets of FOXM1. A similar gene expression profile to that of FOXM1 was found for PLK1 (Figure 5C), CCNB1 (Figure 5D), CDC25A (Figure 5E), and CENPA (Figure 5F). E2 treatment increased the expression of these genes, and cotreatment with Api antagonized the effects of E2. As observed for FOXM1 expression, the inhibitory effect of Api on the expression of the PLK1 and CCNB1 genes was stronger in the MCF-7/Akt cells than in the MCF-7/Ctrl cells. The results observed for the mRNA expression of CCNB1 were similar to those observed for the protein expression of CCNB1 presented in Figure 2G. Finally, as shown in Figure 5G, treatment with E2 reduced the expression of p21 by approximately two-fold. Conversely, treatment with Api increased p21 expression, and this effect was markedly observed in the MCF-7/Akt cells.

Overall, our results show that apigenin alone or in combination with E2 inhibits the expression of FOXM1, which plays an important role in the antiproliferative effects of Api in MCF-7/Akt cells.

## 3. Discussion

Targeted cancer therapies are drugs that inhibit specific molecular targets that are responsible for enhanced tumor growth [40]. In BC, the hyperactivation of Akt is associated with resistance to endocrine therapies [7,8]. However, to date, no drugs that target Akt have been approved for clinical use due to their limited efficiency or toxicity [40]. Targeting signaling pathways whose activities are stimulated in BC tumors with overactivated Akt is one of the strategies for combatting this disease. In this study, we focused on FOXO3a/FOXM1 signaling, which is regulated by Akt [14].

Api, an edible plant-derived flavonoid, has attracted attention because of its anticancer effects, as shown in several experimental and biological studies [41]. Api leads to cell growth arrest and apoptosis in different tumor types by modulating several signaling pathways [41]. As a potential treatment or adjuvant for endocrine-resistant BC [32,33,34], more studies are needed to elucidate the mechanisms of action of Api. To the best of our knowledge, our study is the first to investigate the effect of Api in breast cancer cells with activated Akt.

In this study, we first established MCF-7 cells by which Akt was constitutively active. This approach allowed us to compare the effect of Api on the proliferation of these cells and that of the parental MCF-7/Ctrl cells. As we have previously reported in different ER-positive BC cells [42], Api also exerted a partial antagonistic effect on MCF-7/Ctrl cell proliferation. However, Api exerted a completely antagonistic effect in MCF-7/Akt breast cancer cells. This discrepancy may be explained by the action of Api in signaling pathways that are strongly activated and can promote the proliferation of MCF-7/Akt cells.

Furthermore, we reported that MCF-7/Akt cells were resistant to 4-OHT treatment and that Api partially reduced this resistance by enhancing the anti-proliferative effect of 4-OHT. These observations are consistent with other studies that have shown that Api can inhibit drug resistance mechanisms and improve efficacy [32,33,34,35]. Liu et al. showed that Api increases cytotoxic effects of cisplatin through p53-modulated apoptosis [32]. In adriamycin-resistant breast cancer cells, Api overcomes drug resistance by blocking the signal transducer and activator of transcription 3 [33]. Moreover, Api exerts inhibitory effects on breast cancer resistance protein (BCRP) and decreases the BCRP-mediated efflux of doxorubicin and temozolomide [34]. Recently, Sudhakaran et al. showed that Api sensitizes triple-negative BC spheroids to doxorubicin by targeting hnRNPA2 [35]. Notably, our study is the first to investigate the combination of 4-OHT and Api in the context of BC cells with activated Akt.

The next step of our study was to better understand the mechanisms involved in the effects of Api on breast cancer cell proliferation. First, with respect to ER signaling, Api exerted agonist/antagonist effects on some ER target genes, but complete agonist effects on others. This observation can be explained by the fact that Api exerted agonist/antagonist effects on the activation of ER signaling via AP1 and SP1 but exerted complete agonist effects via the ERE. These effects on ER signaling can explain the agonist/antagonist effects of Api on MCF-7/Ctrl cell proliferation and the antagonist effect of Api when administered with E2 in MCF-7/Akt cells. However, the effects of Api on ER activity cannot explain the antiproliferative effects of treatment with Api alone on the MCF-7/Akt clone. The anti-proliferative effects of Api on MCF-7/Akt cells may act through other signaling pathways.

One explanation for the resistance of our MCF-7/Akt cells to 4-OHT treatment could be the increased expression of FOXM1. The correlation between elevated FOXM1 expression and endocrine resistance in breast tumors has previously been well-demonstrated [12]. Indeed, the FOXM1 oncogene is positively regulated by both ER and PI3K/Akt/FOXO3a signaling [14]. Akt negatively regulates the FOXO3a protein by inducing its exclusion from the nucleus and presence in the cytoplasm, where FOXO3a undergoes degradation [14]. Consistently, in the MCF-7/Akt cells, in which Akt was constitutively activated, we observed a drastic decrease in the FOXO3a protein levels and in FOXO3a translocation from the nucleus to the cytoplasm. We also observed that the FOXO1 proteins levels are clearly lower in the MCF-7/Akt cells than in the MCF-7/Ctrl cells (Supplementary Figure S3). Consequently, the level of FOXM1, which is negatively regulated by the FOXOs proteins, was clearly increased in the MCF-7/Akt cells compared to the MCF-7/Ctrl cells.

We observed no clear effects of Api on the expression or nuclear/cytoplasmic localization of FOXO3a (data not shown). Regarding the FOXO1 protein, there is a slight but not significant increase of its expression under Api treatment in MCF-7/Akt cells (Supplementary Figure S3). However, it is interesting to note that Api acts at the levels of both FOXM1 expression and activity. Api treatment decreased FOXM1 expression at both the gene and protein levels in the MCF-7/Akt cells, and the level of FOXM1 was higher in the MCF-7/Akt cells than in the MCF-7/Ctrl cells. Moreover, Api treatment also decreased the E2-induced stimulation of FOXM1 expression in the two clones. A similar profile was found in FOXM1-related genes involved in the cell cycle. In particular, the inhibitory effects of Api on cyclin B1, which is a protein that is essential for the G2/M transition, can explain the anti-proliferative effects of Api. Api treatment increased cell accumulation in the G2/M phases, but in the absence of cyclin B1, the cells did not have the necessary energy to transition from the G2 phase to the M phase.

Consequently, in response to Api treatment, the cells were arrested at the G2 phase. Our results regarding the effect of Api on the expression of FOXM1 are similar to the recently reported results obtained with compound NB-73, which is an inhibitor of FOXM1 [43]. In that study, Ziegler et al. showed that NB-73 decreased the expression of FOXM1 and simultaneously increased the accumulation of cells in the G2/M phases. Furthermore, the induction of G2/M phase cell cycle arrest by Api in MCF-7 cells was also recently documented by Shendge et al. [44]. According to those authors, this effect of Api is due to its induction of intracellular ROS production. Our findings regarding the action of Api through FOXM1 signaling are complementary to the findings regarding the effects of Api on the cell cycle.

The alteration in the cell cycle that caused the Api-induced G2/M phase cell cycle arrest may result in cell apoptosis. As expected, we showed that Api induced apoptosis in both the MCF-7/Ctrl cells and MCF-7/Akt cells. Our results were consistent with the results of other previous studies, in which Api was shown to induce apoptosis in different cancer cells, such as BC [24,25,26,27,28,35], ovarian cancer [45], and lung cancer [46]. Api exerts apoptotic effects through increases in the expression of p53 and the ratio of Bax/Bcl2, activation of caspases and cleavage of PARP [44,45,46]. Since the cell number is the result of cell proliferation, survival and cell death, the apoptotic effects of Api, accompanied by our results regarding the cell cycle, can explain the anti-proliferative effects of Api observed in our cell proliferation assays.

Together, in this study, we showed anti-proliferative effects of Api in Akt-activated MCF-7 breast cancer cells. The mechanisms of action of Api through ER signaling and Akt/FOXO3a/FOXM1 signaling were revealed. Notably, we observed a clear inhibitory effect of Api on FOXM1 and FOXM1 activity. Since FOXM1 is upregulated and overexpressed in aggressive therapy-resistant forms of breast cancer and is associated with worse prognosis of patients [43,47], Api warrants further study as a potential treatment for endocrine-resistant BC.

## 4. Materials and Methods

### 4.1. Reagents

Apigenin (Api) (A3145) ≥97% (TLC) from parsley, 4-hydroxytamoxifen (4-OHT) (H7904) ≥98% Z isomer, and β-estradiol (E2) (E8875) ≥98% were purchased from Sigma-Aldrich (St Louis, MO, USA).

### 4.2. Cell Clone Establishment and Maintenance

The MCF-7 subclones were established using the T-RexTM system (Thermo Fisher Scientific, Waltham, MA, USA), including the pcDNA6/TR and pcDNA4/TO plasmids. Ctrl and Akt pcDNA4/TO expression vectors were generated from the empty vector and myr-Akt1 cDNA (activated) vector pUSEamp (Upstate Cell Signaling Solutions, Lake Placid, NY, USA), respectively. Note that we had to mutate the PmeI site of the pcDN4/TO plasmid to insert the myr-Akt1 cDNA vector. In detail, one of the two PmeI sites of the pcDN4/TO plasmid was changed into a Hpal site by a direct mutation, and Myr-Akt1 cDNA was inserted between the Hpal and HindIII sites. Stably transfected MCF-7/Ctrl and MCF-7/Akt clones were obtained by transfecting MCF-7 cells with a pcDNA6/TR plasmid and corresponding pcDNA4/TO expression vectors with the jetPEI reagent (Polyplus Transfection, Illkrich, France). Zeocin (75 µg/mL; Thermo Fisher Scientific, Waltham, MA, USA) and blasticidin (5 µg/mL; Thermo Fisher Scientific, Waltham, MA, USA) were used as the selection antibiotics. The stable clones were maintained in Dulbecco’s modified Eagle’s medium (DMEM, Thermo Fisher Scientific, Waltham, MA, USA) supplemented with 4.5 g/L glucose, nonessential amino acids (NEAA, Thermo Fisher Scientific, Waltham, MA, USA), penicillin/streptomycin (Thermo Fisher Scientific, Waltham, MA, USA), 8% fetal bovine serum (FBS, Biowest, Nuaillé, France) and the two selective antibiotics. All the cells were cultured at 37 °C in 5% CO_2_. Before treatment, the cells were cultured in serum-free DMEM without phenol red or steroids and with 2% charcoal/dextran-stripping FBS (Biowest, Nuaillé, France) and treated with 1 µg/mL tetracycline to activate Akt.

### 4.3. Proliferation Assay

Cell proliferation was tested using the sulforhodamine B assay. A total of 3000 cells/well were seeded into 96-well plates. After 24 h, the cells were incubated with serum- and steroid-deficient medium and treated with 1 µg/mL tetracycline for 48 h. The cells were treated with solvent (0.1% DMSO (*v*/*v*) and 0.1% ethanol (*v*/*v*)) as a control, 4-OHT, 10 µM Api alone or in combination with 4-OHT, in the presence or absence of 1 nM E2 for 6 days. At the end of the experiment, the cells were fixed using trichloroacetic acid (TCA) (T0699, Sigma-Aldrich, St Louis, MO, USA) for 1 h at 4 °C and then washed 4 times with tap water. After air drying, the cells were stained with 0.04% (*w*/*v*) sulforhodamine B for 30 min and then washed 4 times with 1% (*v*/*v*) acetic acid. After air-drying one more time, the protein-bound dye was solubilized using the Tris base solution at 10 mM. The absorbance was measured at 490 nm. The cell numbers were determined using calibration ranges (Supplementary Figure S1).

### 4.4. Cell Cycle Analysis by Flow Cytometry (FACS)

A total of 1,000,000 cells per dish were plated in 10-cm dishes and incubated for 24 h. Then, the cells were deprived of steroids and serum for 24 h. Next, the cells were treated with solvent (0.1% DMSO (*v*/*v*) and 0.1% ethanol (*v*/*v*)) as a control, 4-OHT, 10 µM Api alone or in combination with 4-OHT, in the presence or absence of 1 nM E2 for approximately 24 h. After treatment, the cells were trypsinized and fixed with 70% ethanol before being stained with propidium iodide (Sigma-Aldrich, St Louis, MO, USA) in the presence of RNase A. The cell cycle was analyzed with a FACSCalibur flow cytometer (BD Biosciences, Franklin Lakes, NJ, USA).

### 4.5. Apoptosis Analysis

A total of 20,000 cells/well were grown on 10-mm-diameter coverslips in 24-well plates. After 24 h, the cells were incubated with serum- and steroid-deficient medium and treated with 1 µg/mL tetracycline for 48 h. The cells were treated with solvent (0.1% DMSO (*v*/*v*) and 0.1% ethanol (*v*/*v*)) as a control, 4-OHT, 10 µM Api alone or in combination with 4-OHT, in the presence or absence of 1 nM E2 for approximately 24 h. Terminal deoxynucleotidyl dUTP nick end labeling (TUNEL) staining was performed with an In Situ Cell Death Detection Kit, Fluorescein (Sigma-Aldrich, St Louis, MO, USA) according to the manufacturer’s instructions. The percentage of TUNEL-positive cells was determined by using ImageJ software (NIH, Bethesda, MD, USA).

### 4.6. Protein Extraction and Western Blotting

A total of 300,000 cells/well were seeded into 6-well plates. After 24 h, the cells were incubated with serum- and steroid-deficient medium and treated with 1 µg/mL tetracycline for 48 h. Then, the cells were treated with solvent (0.1% DMSO (*v*/*v*) and 0.1% ethanol (*v*/*v*)) as a control or 10 µM Api alone or in combination with 1 nM E2 for 24 h. After the treatment, the cells were directly lysed in 2X Laemmli buffer. The protein extracts were denatured for 10 min at 95 °C, separated on 7.5% SDS polyacrylamide gels, and transferred to polyvinylidene difluoride membranes (Millipore). The proteins were then probed with specific antibodies. The following antibodies were used: anti-Akt1 (sc-5298, Santa Cruz Biotechnology, Dallas, TX, USA) (dilution 1:1000), anti-FOXM1 (D12D5, Cell Signaling Technology, Danvers, MA, USA) (dilution 1:1000), anti-FOXO3a (ab53287, Cambridge, MA, USA) (dilution 1:1000), anti-cyclin B1 (sc-245, Santa Cruz Biotechnology, Dallas, TX, USA) (dilution 1:1000), anti-FOXO1 (C29H4, Cell Signaling Technology, Danvers, MA, USA) (dilution 1:1000) and anti-β-actin (A1978, Sigma-Aldrich, St Louis, MO, USA) (dilution 1:5000), which served as a control for the total amount of protein. An enhanced chemiluminescence system (Immune-Star, Bio-Rad, Hercules, CA, USA) was used to detect the immunocomplexes.

### 4.7. Luciferase Assay

A total of 40,000 cells/well were seeded into 24-well plates. After 24 h, the cells were incubated with serum- and steroid-deficient medium and treated with 1 µg/mL tetracycline for 48 h. The cells were transfected using JetPEI (Polyplus Transfection) overnight with an artificial promoter containing an ERE upstream of the TK promoter (ERE-Luc), AP1-Luciferase (AP1-Luc), or Sp1-Luciferase (Sp1-Luc). As a control for the transfection efficiency, a CMV-β galactosidase vector was used. The following day, the cells were treated with solvent (0.1% DMSO (*v*/*v*) and 0.1% ethanol (*v*/*v*)) as a control or 10 µM Api, alone or in combination with 1 nM E2 for 24 h. Then, reporter lysis buffer (Promega, Madison, WI, USA) was used to lyse the cells, and the luciferase activity was measured with a commercial luciferase assay system (Promega, Madison, WI, USA). The luciferase activity was normalized to β-galactosidase activity. The data are presented as the fold change relative to the control condition.

### 4.8. Immunofluorescence

A total of 20,000 cells/well were grown on 10-mm-diameter coverslips in 24-well plates. After 24 h, the cells were incubated with serum- and steroid-deficient medium and treated with 1 µg/mL tetracycline for 48 h. Then, the cells were treated with solvent (0.1% DMSO (*v*/*v*) and 0.1% ethanol (*v*/*v*)) as a control and 10 µM Api alone or in combination with 1 nM E2 for 24 h. After treatment, the cells were fixed with 4% formaldehyde solution/neutral buffered saline (Sigma-Aldrich, St Louis, MO, USA) for 15 min and then permeabilized in PBS/3% Triton X-100 for 10 min. Next, the cells were unmasked with 50 mM Tris-HCl pH 9.5 at 80 °C for 30 min. The cells were incubated overnight at 4 °C with primary antibodies against phospho-Bad (Ser112, Cell Signaling Technology, Danvers, MA, USA) (dilution 1:1000), phospho-mTOR (Ser2448, Cell Signaling Technology, Danvers, MA, USA) (dilution 1:1000), FOXO3a (ab124394, Abcam, Cambridge, MA, USA) (dilution 1:1000) or FOXM1 (D12D5, Cell Signaling Technology, Danvers, MA, USA) (dilution 1:1000) and then with a secondary antibody (dilution 1:1000) at room temperature for one hour. The coverslips were mounted with Duolink^®^ in situ mounting medium with 4′,6-diamidino-2-phenylindole (DAPI) (Sigma-Aldrich, St Louis, MO, USA) before microscopic analysis.

### 4.9. Nuclear/Cytoplasmic Ratio Quantification from Immunofluorescence Images

ImageJ software (NIH, Bethesda, MD, USA) was used to create a custom macro to generate an automated tool to quantify the nuclear/cytoplasmic fluorescence ratio of each cell in the images. The first mask, corresponding to the nuclear area, was determined using DAPI fluorescence. The second mask, which was considered to be the cytoplasmic area, is created by homothetic enlargement of the first mask to generate a ring around the nucleus. These two masks were successively applied to quantify the fluorescence of the protein of interest in the nucleus and in the cytoplasmic area. Then, the nuclear/cytoplasmic immunofluorescence ratio was calculated for each cell. The median of the nuclear/cytoplasmic ratio of the control condition was used as a threshold to determine the percentage of cells below and above this threshold in each condition. For each condition, approximately 1000–1500 cells from at least 10 images were analyzed.

### 4.10. RNA Extraction and Real-Time PCR

A total of 300,000 cells/well were plated in 6-well plates. After 48 h of serum and steroid deprivation and tetracycline treatment, the cells were treated with solvent (0.1% DMSO (*v*/*v*) and 0.1% ethanol (*v*/*v*)) as a control and 10 µM Api alone, or in combination with, 1 nM E2 for 24 h. The total RNA was obtained using a mini RNeasy kit (Qiagen, Hilden, Germany) following the manufacturer’s instructions. Then, using an M-MLV RT kit (Thermo Fisher Scientific, Waltham, MA, USA), the RNA was reverse-transcribed according to the manufacturer’s instructions. For real-time PCR, 5 ng of cDNA was used with 150 nM primers (Table 1) and iTaq Universal SYBR Green Supermix (Bio-Rad, Hercules, CA, USA). Real-time PCR was carried out in a CFX 384 apparatus, and the data were analyzed with CFX Manager Software (Bio-Rad, Hercules, CA, USA).

### 4.11. Statistical Analyses

Statistical significance was quantified with one-way analysis of variance (ANOVA) and Tukey’s post hoc test or with a Mann-Whitney test using GraphPad Prism software (version 5, GraphPad Software, San Diego, CA, USA).

## Figures and Tables

**Table 1:** Gene names and primer sequences used in this study.

Gene Name and Symbol	Forward Primer	Reverse Primer
GATA-binding protein 3 (GATA3)	GCCGTTGAGGGTTTCAGAGA	TCCGAGCACAACCACCTTAG
Forkhead box A1 (FOXA1)	CCCCTTTGTCCTCTCTACCC	CTGCAAAGCAAGAAGCAGAGT
Estrogen receptor 1 (ESR1/ERα)	TTTATGGGAAAAGGCTCAAA	GACAAAACCGAGTCACATCA
Chemokine (C-X-C motif) ligand 12 (CXCL12)	CACCATTGAGAGGTCGGAAG	AATGAGACCCGTCTTTGCAG
Progesterone receptor (PgR)	CCCGCCGTCGTAACTTTGG	GTGCCTATCCTGCCTCTCAATC
Amphiregulin (AREG)	GTATTTTCACTTTCCGTCTTGTTTTG	CCTGGCTATATTGTCGATTCA
Forkhead box M1 (FOXM1)	AGTGCCAACCGCTACTTGAC	CACCGGGAACTGGATAGGTA
Cell division cycle 25A (CDC25A)	CAAGGGTGCAGTGAACTTGC	ACAACAATGACACGCTTGCC
Cyclin B1 (CCNB1)	TCTGGATAATGGTGAATGGACA	CGATGTGGCATACTTGTTCTTG
Centromere protein A (CENPA)	ACATGCAGGCCGAGTTACTC	AGAGTCCCCGGTATCATCCC
Polo-like kinase 1 (PLK1)	CTCAACACGCCTCATCCTC	GTGCTCGCTCATGTAATTGC
Cyclin-Dependent kinase inhibitor 1A (CDKN1A/p21)	CTGTCTTGTACCCTTGTGCC	GGTAGAAATCTGTCATGCTGG
Glyceraldehyde-3-phosphate dehydrogenase (GAPDH)	TGCACCACCAACTGCTTAGC	GGCATGGACTGTGGTCATGAG
TATA box-binding protein (TBP)	TGCACAGGAGCCAAGAGTGAA	CACATCACAGCTCCCCACCA

## Data Availability

The data presented in this study are available on request from the corresponding author. The data are not publicly available due to privacy.

## Supplementary Materials

The following are available online at https://www.mdpi.com/1422-0067/22/1/470/s1, Figure S1: Calibration ranges of cell number and absorbance tests by sulforhodamine B assay, Figure S2: Nuclear/cytoplasmic fluorescence intensity ratio of FOXM1, Figure S3: Equal amounts of protein extracts from MCF-7/Ctrl cells and MCF-7/Akt cells were analyzed by Western blotting with antibodies specific for FOXO1 and β-actin.

## Abbreviations

4-OHT	4-Hydroxytamoxifen
Api	Apigenin
BC	Breast cancer
E2	Estradiol
FOXM1	Forkhead box protein M1
TNBC	Triple-negative breast cancer
