# Supplementary material for: Apigenin, a Partial Antagonist of the Estrogen Receptor (ER), Inhibits ER-Positive Breast Cancer Cell Proliferation through Akt/FOXM1 Signaling
Source: Int J Mol Sci. 2021 Jan 5;22(1):470. doi: 10.3390/ijms22010470 (PMC7796491; doi:10.3390/ijms22010470)
Supplement: Supplementary file 1 [file ijms-22-00470-s001.pdf]

Supplementary Figure S1

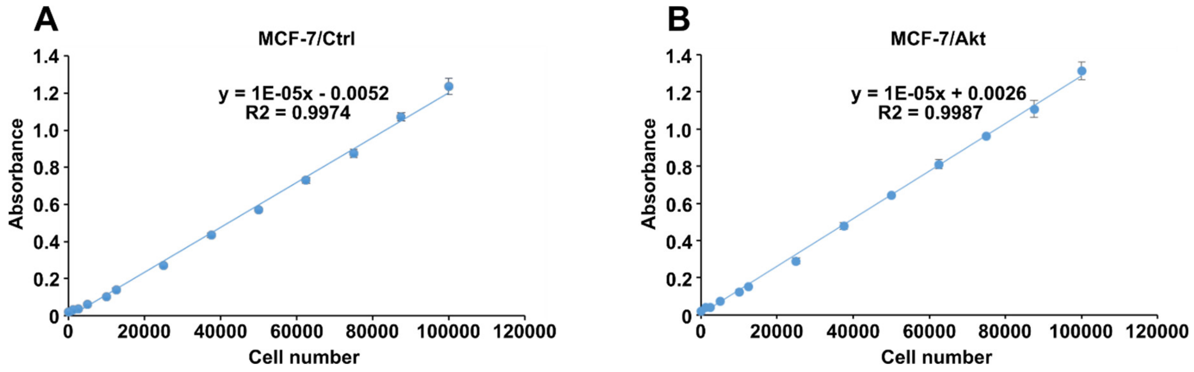

Supplementary Figure S1. Calibration ranges of cell number and absorbance tests by sulforhodamine B assay. MCF-7/Ctrl cells (A) and MCF-7/Akt cells (B) were seeded into 96-well plates and incubated for 6 h. Then, the cells were fixed and stained with sulforhodamine B. The absorbance of the protein-bound dye was measured at 490 nm. The results showed that the relation between cell number and absorbance followed a linear function. The linear equation and coefficient of determination are indicated in each graph.

Supplementary Figure S2

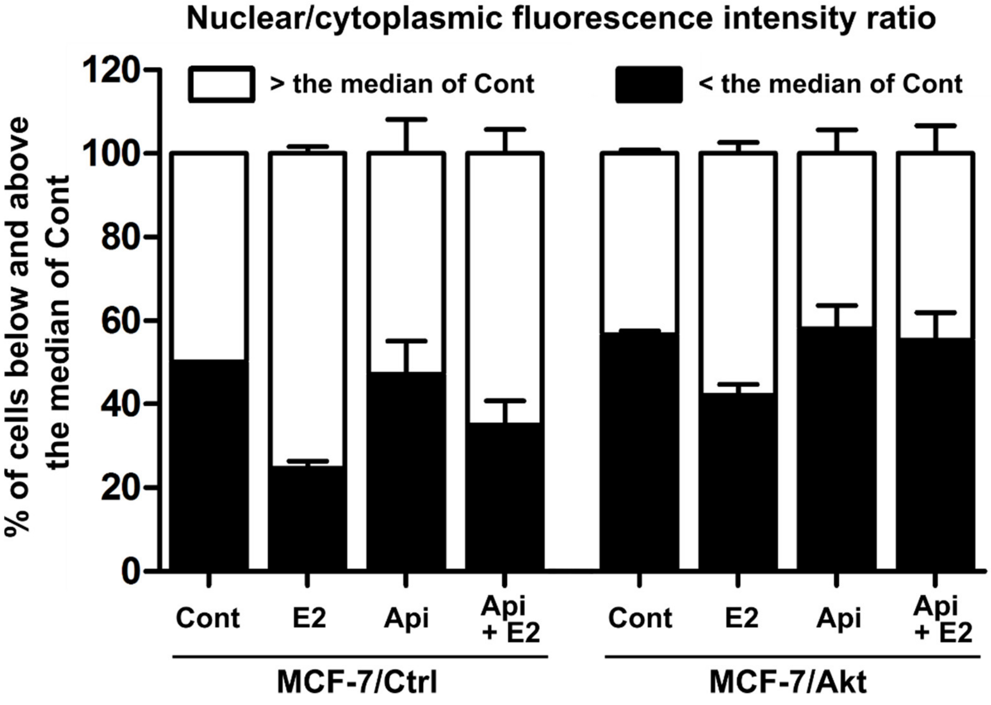

Supplementary Figure S2. Nuclear/cytoplasmic fluorescence intensity ratio of FOXM1. MCF-7/Ctrl cells and MCF-7/Akt cells were treated with 0.1% (v/v) DMSO and 0.1% (v/v) ethanol as the control (Cont), 1 nM estradiol (E2), or 10 µM apigenin (Api) alone or in combination with E2 for 24 h. The fluorescence intensities in the nucleus and cytoplasm of each cell was measured using ImageJ software. The median of the nuclear/cytoplasmic ratio of the control condition was used as a threshold to determine the percentage of cells below and above this threshold in each condition. For each condition, approximately 1000-1500 cells from at least 10 images were analyzed. The experiment was conducted 3 times. The data are presented as the mean ± SEM.

Supplementary Figure S3

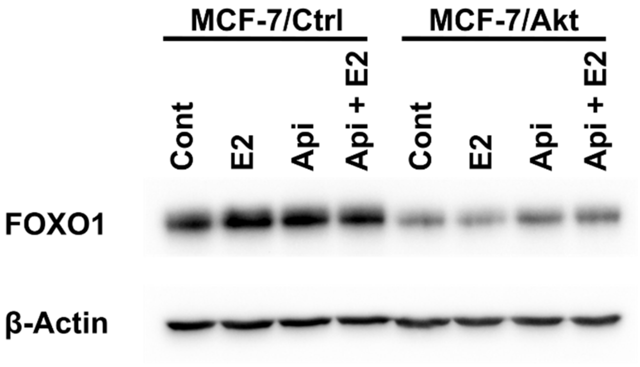

Supplementary Figure S3. Equal amounts of protein extracts from MCF-7/Ctrl cells and MCF-7/Akt cells were analyzed by Western blotting with antibodies specific for FOXO1 and β-actin. MCF-7/Ctrl cells and MCF-7/Akt cells were treated with solvent (Cont), E2 and Api alone or in combination with E2 for 24 h
